# Cerebral Arterial Gas Embolism due to Helium Inhalation from a High-Pressure Gas Cylinder

**DOI:** 10.1155/2022/1847605

**Published:** 2022-03-08

**Authors:** Gabriel Morales, Marie Fiero, Jesselle Albert, Jane Di Gennaro, Anthony Gerbino

**Affiliations:** ^1^Department of Emergency Medicine, University of Washington, Seattle, WA, USA; ^2^Divsion of Pediatric Critical Care Medicine, Seattle Children's Hospital, Seattle, WA, USA; ^3^Sections of Critical Care and Pulmonary Medicine, Virginia Mason Medical Center, Seattle, WA, USA; ^4^Center for Hyperbaric Medicine, Virginia Mason Medical Center, Seattle, WA, USA

## Abstract

Cerebral arterial gas embolism (CAGE) is a rare but serious cause for acute neurologic deficit that occurs most often in divers who breathe compressed gas at depth or iatrogenically from a variety of invasive medical procedures. We present a rare case of CAGE caused by inhaling helium from an unregulated, high-pressure gas cylinder. Following inhalation, the patient experienced loss of consciousness, neurologic deficits, pneumomediastinum, and pneumothorax requiring transfer and treatment at a hyperbaric facility with resulting resolution of neurologic symptoms. This case highlights the importance of rapid diagnosis and hyperbaric oxygen treatment (HBO), facilitated by close coordination among community emergency departments, pediatric tertiary care centers, hyperbaric facilities, and poison control.

## 1. Introduction

Cerebral arterial gas embolism (CAGE) occurs most often in divers who breathe compressed gas at depth or iatrogenically from a variety of invasive medical procedures [1–4]. We present a rare case of CAGE caused by inhaling helium from an unregulated, high-pressure gas cylinder. The frequency of simar events is unknown but may be more common than reported. Timely recognition is critical for optimal care. Hyperbaric oxygen treatment (HBO), the primary treatment for CAGE, acts to improve tissue oxygenation and provide anti-inflammatory effects [5–8]. As decreased time to HBO results in improved outcomes [4], this case highlights the importance of rapid diagnosis, transport, and implementation of HBO therapy, facilitated by close coordination among community emergency departments, pediatric tertiary care centers, hyperbaric facilities, and poison control.

## 2. Case Report

A previously healthy 12-year-old female presented to a community emergency department (ED) after a single inhalation of helium gas from a high-pressure gas cylinder purchased from a national retail store. Within a few seconds of inhaling helium, she began coughing and then quickly lost consciousness for less than one minute. Upon awakening, the patient was disoriented and complained of headache, chest pain, visual deficits, and weakness. She was unable to ambulate due to right-sided weakness.

She was transported to a nearby ED where her vital signs, oxygen saturation, cardiac, and lung exam were normal except for sinus tachycardia. She was awake, alert, and oriented, with improved mentation since the inhalation event. Her right arm was objectively weak but able to move against gravity but she could not independently lift her right leg off the bed. Additionally, she had dysarthria and a right-sided facial droop.

**Figure 1:**
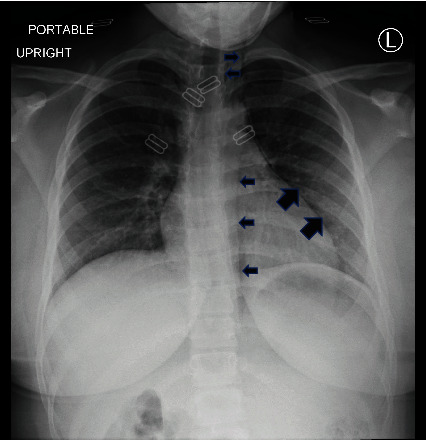
Chest X-ray upon admission to the emergency department demonstrates pneumomediastinum without pneumothorax (solid arrows).

**Figure 2:**
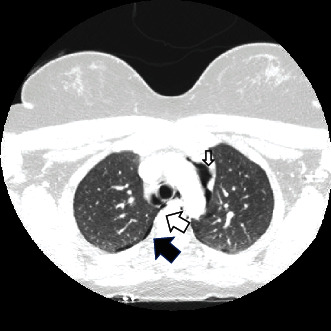
CT angiography of the head and neck. Small right apical pneumothorax (solid arrow) and pneumomediastinum (nonsolid arrows) are demonstrated.

Chest X-ray revealed pneumomediastinum and subcutaneous emphysema of the neck (Figure 1). CT of the brain without contrast was normal. CT angiogram of the head and neck (Figure 2) was notable for a small right apical pneumothorax and subcutaneous emphysema but no abnormalities within the vasculature or brain parenchyma. EKG demonstrated sinus rhythm. Creatine kinase was 327, and serum troponin was normal. The case was first discussed with Washington State Poison Control and then the regional hyperbaric and pediatric tertiary medical centers. She was placed on 100% oxygen via a nonrebreather mask and positioned supine. A right-sided chest tube was placed prior to transport given concern for expansion of the pneumothorax during HBO. Due to the lack of pediatric inpatient services at the hyperbaric center, the patient was first flown by fixed-wing airplane utilizing sea level cabin pressure to the regional pediatric tertiary center.

Upon arrival at the pediatric facility, the patient had persistent right arm and leg weakness with right facial droop, loss of right leg pinprick sensation, and dysarthria. She continued to be alert and oriented with GCS 15. Visual acuity was normal. After rapid assessment in the pediatric intensive care unit (PICU) to ensure stability and placement of arterial catheter for monitoring during HBO treatment, the patient was then transferred to the nearby hyperbaric center by ambulance accompanied by both a PICU physician and a PICU nurse. HBO began 6 hours, 45 minutes following helium inhalation. She received a total of 6 hours, 10 minutes of HBO according to US Navy Treatment Table 6, the standard protocol for arterial gas embolism (AGE). Complete resolution of neurologic deficits was noted after 90 minutes of HBO. The patient returned to the PICU for close neurologic monitoring. Her subsequent neurologic exam varied by provider between normal and minimal deficits; therefore, no additional HBO was given. An MRI and MRA of the brain and a transthoracic echocardiogram with bubble study were normal on hospital day 3, the day of discharge.

## 3. Discussion

We describe a case of pulmonary barotrauma and CAGE resulting in neurological deficits as a result of inhaling helium from an unregulated, high-pressure gas cylinder. The inhalation of helium under high pressure resulted in disruption of the alveolar interface with dissection of gas into the pulmonary venous circulation [9] as evidenced by the development of pulmonary barotrauma. The pressure in a retail helium gas cylinder is approximately 13,500 mm Hg (1800 kPA), greatly exceeding the transpulmonary pressure of 60-80 mm Hg (8.0-10.7 kPa) required to rupture alveoli [10]. The presence of pulmonary barotrauma, abrupt loss of consciousness, and development of focal neurologic deficits after a single inhalation of helium, elevation in creatine kinase [4, 11], and rapid response to hyperbaric oxygen all support the diagnosis of CAGE.

The frequency of CAGE from high-pressure helium inhalation is unknown. There have been no reports in the medical literature since 2002, with three reports including one pediatric patient prior to that time [9, 12, 13]. Two reports in the lay press in 2012 and 2015 detail helium inhalation in children leading to death and disability, respectively, due to CAGE [14, 15]. Helium is most often inhaled by adolescents to recreationally change voice quality, but use has also been described for its possible intoxicating effects [16]. Numerous Internet sources demonstrate helium inhalation from balloons or pressurized gas cylinders. The wide availability of high-pressure helium tanks from retail stores and the encouragement of “helium huffing” on social media raise concern that helium-related CAGE may be more common than reports suggest. This mechanism should be distinguished from helium-induced asphyxia caused by continuously breathing helium under low pressure [17, 18], usually in an attempt at suicide, but occasionally by accident in the pediatric population [19, 20].

Diagnosis of CAGE is primarily clinical, including stroke-like symptoms, loss of consciousness, or death during or immediately after an at-risk activity or procedure. Because all previously reported cases of CAGE due to helium inhalation describe immediate loss of consciousness and pulmonary barotrauma [9, 12–15], the diagnosis of CAGE should be entertained in anyone losing consciousness or with pulmonary barotrauma shortly after high-pressure gas inhalation. However, evidence of pulmonary barotrauma is often absent in divers with AGE or CAGE [21, 22], and its absence should not exclude the diagnosis.

Additional imaging studies are often obtained when CAGE is suspected but are not sensitive enough to exclude the diagnosis and should not materially delay HBO. The presence of gas within cerebral vasculature or brain parenchyma confirms the diagnosis, while gas within cardiac chambers or great vessels strongly supports the diagnosis [23]. However, these findings are frequently absent and are not required to make a diagnosis of CAGE [4, 9, 23–25]. Imaging was utilized in this case given the unusual mechanism of injury and immediate availability of CT to assess for other acute intracranial pathology during the time to arrange transportation to the pediatric facility.

HBO is the primary treatment for CAGE [26] with shorter delays to treatment associated with improved outcomes [4, 27]. HBO decreases the volume of gas bubbles via Boyle's Law, increases blood and tissue oxygenation, and has anti-inflammatory effects that confer neuroprotection via multiple mechanisms [5–8]. While early HBO is ideal, delays up to 24 hours should not exclude therapy, as it may still be beneficial [3, 27, 28] due to HBO's anti-inflammatory effects. Prior to HBO, patients should be placed on high-flow oxygen and positioned supine. Animal models of CAGE suggest that lidocaine may improve neurologic function [13, 29], but we did not administer lidocaine given the lack of human data and possible side effects such as seizures.

Diagnosis and treatment of CAGE in children present several challenges. Lack of familiarity with helium inhalation causing CAGE may delay diagnosis. The vast majority of hyperbaric centers do not treat patients requiring emergent treatment, therefore lengthening travel time to facilities that do [30, 31]. Emergency hyperbaric centers may lack pediatric services requiring collaboration between pediatric and hyperbaric centers in the care of critically ill children. Our patient benefited from preexisting relationships between the community hospital, poison control, regional hyperbaric, and pediatric medical centers. Such institutional relationships may help facilitate rapid diagnosis and transfer to safe, definitive care.

We have presented a case of CAGE due to inhalation of helium gas from an unregulated, high-pressure gas cylinder that resulted in pulmonary barotrauma. Immediate loss of consciousness or focal neurologic deficits after high-pressure gas inhalation should trigger consideration of CAGE. Rapid diagnosis and HBO treatment were facilitated by close coordination among community emergency departments, pediatric tertiary care centers, hyperbaric facilities, and poison control.

## Data Availability

Data sharing not applicable to this article as no datasets were generated or analyzed during the current study.
